# Enactive interventions can enhance agency, health, and social relationships during childhood

**DOI:** 10.3389/fpsyg.2023.1245883

**Published:** 2024-01-03

**Authors:** Mariana Lozada, Paola D'Adamo

**Affiliations:** ^1^INIBIOMA, CONICET - Universidad Nacional del Comahue, San Carlos de Bariloche, Argentina; ^2^ECyC IPEHCS CONICET - Universidad Nacional del Comahue, San Carlos de Bariloche, Argentina

**Keywords:** enaction, agency, health, childhood, sociality

## Introduction

Health is a profound, mysterious and complex phenomenon dynamically rooted in the experience of being alive. According to the enactive approach, life involves autonomous self-organizing processes that underlie the intricate relationship between an organism and its environment. In this way, organisms don't merely react to external disturbances by displaying specific actions in a given situation; instead, they actively regulate the conditions of their interaction with the environment. Through this active regulation, they bring forth a world, renewing and sustaining their own constitution (Varela et al., 1991). This approach considers that self-organization entails continuous homeodynamic organismic regulation based on operational closure of the system, from which internal (autonomous) networks emerge (Maturana and Varela, 1991). This perspective emphasizes the significance of agency, understood as the active role played by living beings not only in bringing forth their world but also in creating meaning and value through these actions. In other words, agency is not just a matter of directing one's body and actions, but also of actively participating in the construction of one's own reality. By their actions, organisms directly enact a world of meaning (e.g., Di Paolo, 2021).

In line with this, the enactive approach acknowledges that individuals play an active role in their own health rather than being merely passive entities. Increasing evidence indicates that health processes are traversed by personal histories, motivations, concerns, perception-action patterns and active engagement with the environment (e.g., Kirmayer and Gómez-Carrillo, 2019; Arandia and Di Paolo, 2021; Svenaeus, 2022). As these authors suggest, disruptions in health can be related to limitations in our capacity to be active agents. A plausible scenario in which agency related to our own health can be fostered might be associated with how we handle chronic stress. Regarding potential health disruptions in children, there is growing concern about the rising incidence of chronic stress (e.g. Wehbe et al., 2022). Numerous studies indicate that children may experience chronic stress as a result of diverse factors such as family challenges, economic hardship, traumatic experiences, and exposure to unfavorable living conditions (Bucci et al., 2016; Crielaard et al., 2021; Mousikou et al., 2023).

Chronic stress is a psychological and physiological response to ongoing pressure, tension, or difficulties experienced over an extended period (e.g., Sapolsky, 2004). The prolongation of the stress response over time is a probable contributor to negative health outcomes, mainly due to neuroendocrine dysregulation and long-term inflammatory processes (e.g., Mariotti, 2015; McEwen, 2017). Chronic stress has also been related to mental health problems such as anxiety and depression (e.g., Danese and McEwen, 2012; McEwen, 2016, 2017).

From an enactive viewpoint, stress clearly reflects the deep interconnection between mind, body, and socio-cultural-ecological environments. The stress response involves several psycho-physiological reactions, such as deactivation of the hypothalamic-pituitary-adrenocortical (HPA) axis. This leads to the release of cortisol, a hormone secreted in the adrenal cortex. To evaluate stress levels, cortisol concentration can be measured in a non-invasive way by testing either saliva or hair samples (e.g., Aguilar Cordero et al., 2014; Iglesias et al., 2015). Perceived stress self-reports can also be used to assess chronic stress in children (Trianes et al., 2011).

Another important factor for healthy development during childhood relates to the construction of social relationships (e.g., Berkman et al., 2000; Robles, 2021; Smith and Pollak, 2021). The configuration of social networks, along with the quality and quantity of social interactions, hold considerable importance for health and wellbeing, as acknowledged by various researchers (e.g., Cohen, 2004; Hostinar and Gunnar, 2013; Gunnar, 2017). Interestingly, it has been shown that the way children perceive their immediate social relationships correlates with stress biomarkers (Ponzi et al., 2016). In sum, favorable social relationships can contribute significantly to a child's emotional and mental wellbeing, helping them navigate stress and adversity. On the contrary, social isolation or strained relationships may lead to increased stress levels, potentially impacting neuroendocrine processes and immune function (Cacioppo et al., 2015).

Decisions regarding methodology are not impartial, as different methodological approaches may lead to different results or highlight different aspects of a phenomenon. As proposed by Valsiner (2017), the researcher can be considered an active participant in the research process, interacting with the participants and their context. Transformation experiences can be better understood when evaluated by integrating third, second, and first-person methodologies (Varela, 1996; Varela and Shear, 1999). Third-person perspectives in biomedical approaches tend to assess health through impersonal and impartial measurements, whereas approaches like “patient-centered medicine” consider subjective first-person perspectives that contemplate personal perceptions, feelings, and thoughts. The second-person perspective is given by someone who has undergone a similar situation and can comprehend and interpret the first-person experience. Integrating these three perspectives can help transcend reductionist viewpoints that purport the dualistic notion of internal subjective experience vs. external objectivity (Depraz et al., 2003; Svenaeus, 2022). In the studies described below, we highlight the value of considering these three approaches in order to grasp the complexity of the processes taking place as children participated in enactive interventions aimed at diminishing chronic stress and enhancing social integration.

It seems opportune to encourage sociability and agency at an early age to help children deal with life's challenges. In this opinion article we show how enactive interventions can be put into practice during childhood, favoring children's health, empathy, and social wellbeing.

## Enactive interventions in children

There is increasing evidence that the enactive approach is an influential paradigm which is gaining greater recognition within health domains; to our knowledge, however, within this framework few studies have focused on children's health. In this article we revisit prior research on this topic through the perspective of the enactive lens, which highlights the significance of agency, autonomy, embodied interactions, and participatory sense-making. We emphasize the value of transformative processes that can take place in children during enactive interventions, while considering the multidimensional complexity of mind-body-environment connections. Specifically, these enactive interventions consisted of diverse activities that included mind-body integration practices (favoring attentional processes), playful embodied interactions and reflective-sharing instances (fostering empathy and other-oriented awareness) (Lozada et al., 2014a,b; Fuentes et al., 2018a,b; Carro et al., 2020, 2021, 2022, 2023). Some of these instances were propitiated through non-competitive play and self-awareness activities performed in dyads or larger groups, fostering joint attention, coordination and negotiation. These studies showed that participation in these enactive interventions contributed to diminishing chronic stress while enhancing social integration between peers, bringing about processes of transformation in multiple spheres, in both personal and interpersonal situations.

Chronic stress was assessed from a third-person perspective through cortisol concentration, which exhibited a decrease at the end of the interventions, as did the self-reports of perceived stress (Lozada et al., 2014b; Carro et al., 2020, 2021, 2023). The children's experience was evaluated from the first-person perspective through individual interviews during which they mentioned feeling better after performing the mind-body activities. They reported putting the learned activities into practice when feeling upset, afraid, stressed, or in other adverse situations, both during and after the program (Lozada et al., 2014b; Carro et al., 2020). Moreover, many children mentioned having taught these practices to their parents. These findings suggest that the incorporation of perception-action patterns may have favored emotional regulation processes (self-regulatory habits), indicating a greater sense of agency and awareness. The second-person approach was in line with the children's perceptions, as qualitative observations made by the researchers enabled them to identify significant changes in the children's levels of attention, participation and empathic attitudes. Several teachers also noticed an improvement in the children's attention in class becoming calmer and less irritable as the intervention progressed. Notably, the children often asked to perform the learned activities during normal classes. Regarding qualitative analysis, there was significant agreement among researchers in their observations during each encounter; i.e., investigator inter-agreements showed a high level of consensus.

Within the social sphere, significant changes in peer relationships were observed throughout the intervention program. Analysis of the sociograms showed novel bonding in the social network dynamics: groups became more open, favoring social integration and empathy (Lozada et al., 2014a,b; Fuentes et al., 2018a,b; Carro et al., 2020, 2021, 2022, 2023). As the interventions progressed, the children tended to interact with peers who had previously been rejected as playmates. They were also more capable of dealing with conflicts and disagreements during the games, being able to work together to find new ways of interacting. Throughout the interventions, both pre-existing hostilities and tensions arising during play were largely overcome by the children themselves, without adult involvement. Group reflection, which took place at the end of each encounter, showed significant changes over the intervention period. During this instance, the children gradually showed not only greater confidence when sharing their emotions, their likes and dislikes, and their impressions of the lived experience, but also greater willingness to listen to other classmates' perspectives. In the final interviews, many children emphasized that one of the most valuable experiences was the opportunity to engage with peers they had had little or no prior interaction with. Additionally, the teachers noted that the classroom atmosphere had become more harmonious as a result of fewer aggressive interactions. Interestingly, children commented that they used the learned practices during moments of conflict with others, such as when they argued with a friend or sibling, or when their parents reprimanded them. This shows the potential of this kind of intervention to cultivate children's agency and social relationships.

It is noteworthy that the above-mentioned interventions involved diverse participatory sense-making processes. Moreover, the researchers took an active part in the activities with the children, performing the mind-body integration practices, playing, and sharing their experiences during the reflective instances. We believe that this active participation may have encouraged instantiation of an affective environment that helped children develop greater confidence, enabling them to convey their feelings and points of view (Carro et al., 2022). The atmosphere achieved during the enactive interventions may have helped attenuate hierarchical exchanges, thus favoring children's agency and self-confidence.

## Concluding remarks

In this article we aim to demonstrate how enactive interventions can foster a healthy interplay between mind, body, and social environments, evidenced at multiple levels and assessed from first-, second- and third-person perspectives. As proposed by Varela and Shear (1999) in the field of neurophenomenology, third-person perspectives (e.g., cortisol measurements) can be enriched with inquiries about subjective experience (Depraz et al., 2003). Consistent with this, qualitative methodologies used in the described studies allowed in-depth exploration of the complex transformations experienced by participants. It is worthy of note that in addition to chronic stress reduction, children made use of the novel practices, indicating that agency had been developed in this aspect and enabling them to play a more active role in coping with potential challenges. A similar trend was observed in the social domain since children became more agentive in their interactions with others. This agency enhancement in the social dimension may also have played a significant role in promoting children's wellbeing, given the fundamental involvement of affection and socialization in health processes (e.g., Haas et al., 2010; Trevarthen et al., 2018; Carozza and Leong, 2021; Kornienko et al., 2022). In accordance with this, as warmth and acceptance were central to the intervention, it is likely that self-other awareness activities and non-competitive play favored a nurturing atmosphere within the group, promoting participatory sense-making processes.

In sum, in this article we aimed to share our experience with enactive interventions that can enhance children's health and wellbeing through agentive transformative processes. We believe that this kind of embodied experience can serve them well in their daily lives and can be incorporated easily into educational contexts, promoting children's healthy development.

## Author contributions

ML and PD'A were responsible for the design, elaboration and writing of the manuscript, literature review, and data analysis. All authors contributed to the article and approved the submitted version.

## References

[B1] Aguilar Cordero M. J. Sánchez López A. M. Mur Villar N. García García I. López R. Ortegón Piñero A.. (2014). Cortisol salival como indicador de estrés[[Inline Image]] fisiológico en niños y adultos: revisión sistemática. Nutr. Hosp. 29, 960–968. 10.3305/nh.2014.29.5.7273 24951973

[B2] Arandia I. R. Di Paolo E. A. (2021). Placebo from an enactive perspective. Front. Psychol. 12, 660118. 10.3389/fpsyg.2021.660118 34149551 PMC8206487

[B3] Berkman L. F. Glass T. Brissette I. Seeman T. E. (2000). From social integration to health: Durkheim in the new millennium. Soc. Sci. Med. 51, 843–857. 10.1016/S0277-9536(00)00065-4 10972429

[B4] Bucci M. Marques S. S. Oh D. Harris N. B. (2016). Toxic stress in children and adolescents. Adv. Pediatr. 63, 403–428. 10.1016/j.yapd.2016.04.002 27426909

[B5] Cacioppo J. T. Cacioppo S. Capitanio J. P. Cole S. W. (2015). The neuroendocrinology of social isolation. Ann. Rev. Psychol. 66, 733–767. 10.1146/annurev-psych-010814-015240 25148851 PMC5130104

[B6] Carozza S. Leong V. (2021). The role of affectionate caregiver touch in early neurodevelopment and parent–infant interactional synchrony. Front. Neurosci. 14, 613378. 10.3389/fnins.2020.613378 33584178 PMC7873991

[B7] Carro N. D'Adamo P. Lozada M. (2020). An effective intervention can contribute to enhancing social integration while reducing perceived stress in children. Electron. J. Res. Educ. Psychol. 18, 183. 10.25115/ejrep.v18i50.2600

[B8] Carro N. D'Adamo P. Lozada M. (2021). A school intervention helps decrease daily stress while enhancing social integration in children. Behav. Med. 47, 251–258. 10.1080/08964289.2020.1738319 32275198

[B9] Carro N. Ibar C. D'Adamo P. Gonzalez D. Berg G. Fabre B.. (2023). Hair cortisol reduction and social integration enhancement after a mindfulness-based intervention in children. Child Care Health Dev. 49, 73–79. 10.1111/cch.13008 35312189

[B10] Carro N. Kuperman M. D'Adamo P. Lozada M. (2022). Social relationships in children: favourable influence of activities promoting self-awareness and empathic interaction. J. Complex Netw. 10, cnab049. 10.1093/comnet/cnab049

[B11] Cohen S. (2004). Social relationships and health. Am. Psychol. 59, 676. 10.1037/0003-066X.59.8.676 15554821

[B12] Crielaard L. Nicolaou M. Sawyer A. Quax R. Stronks K. (2021). Understanding the impact of exposure to adverse socioeconomic conditions on chronic stress from a complexity science perspective. BMC Med. 19, 1–20. 10.1186/s12916-021-02106-1 34635083 PMC8507143

[B13] Danese A. McEwen B. S. (2012). Adverse childhood experiences, allostasis, allostatic load, and age-related disease. Physiol. Behav. 106, 29–39. 10.1016/j.physbeh.2011.08.019 21888923

[B14] Depraz N. Varela F. J. Vermersch P. (Eds.). (2003). On Becoming Aware: A Pragmatics of Experiencing, Vol. 43. Amsterdam: John Benjamins Publishing. 10.1075/aicr.43

[B15] Di Paolo E. A. (2021). Enactive becoming. Phenomenol. Cogn. Sci. 20, 783–809. 10.1007/s11097-019-09654-1

[B16] Fuentes M. A. Cárdenas J. P. Carro N. Lozada M. (2018a). Development and complex dynamics at school environment. Complexity 2018, 3963061. 10.1155/2018/3963061

[B17] Fuentes M. A. Cárdenas J. P. Carro N. Lozada M. (2018b). Social network plasticity in children. Transylv. Rev. 25, 6679–6686.

[B18] Gunnar M. R. (2017). Social buffering of stress in development: a career perspective. Perspect. Psychol. Sci. 12, 355–373. 10.1177/1745691616680612 28544861 PMC5445937

[B19] Haas S. A. Schaefer D. R. Kornienko O. (2010). Health and the structure of adolescent social networks. J. Health Soc. Behav. 51, 424–439. 10.1177/0022146510386791 21131619

[B20] Hostinar C. E. Gunnar M. R. (2013). The developmental effects of early life stress: an overview of current theoretical frameworks. Curr. Dir. Psychol. Sci. 22, 400–406. 10.1177/0963721413488889 25419054 PMC4236853

[B21] Iglesias S. Jacobsen D. Gonzalez D. Azzara S. Repetto E. M. Jamardo J.. (2015). Hair cortisol: a new tool for evaluating stress in programs of stress management. Life Sci. 141, 188–192. 10.1016/j.lfs.2015.10.006 26454227

[B22] Kirmayer L. J. Gómez-Carrillo A. (2019). Agency, embodiment and enactment in psychosomatic theory and practice. Med. Humanit. 45, 169–182. 10.1136/medhum-2018-011618 31167895 PMC6699606

[B23] Kornienko O. Riis J. Davila M. White N. S. Garner P. W. (2022). Preliminary insights into associations between C-reactive protein and social network dynamics. Psychoneuroendocrinology 139, 105690. 10.1016/j.psyneuen.2022.105690 35193045

[B24] Lozada M. Carro N. D'Adamo P. Barclay C. (2014b). Stress management in children: a pilot study in 7 to 9 year olds. J. Dev. Behav. Pediatr. 35, 144–147. 10.1097/DBP.0000000000000026 24406660

[B25] Lozada M. D'Adamo P. Carro N. (2014a). Plasticity of altruistic behavior in children. J. Moral Educ. 43, 75–88. 10.1080/03057240.2013.878244

[B26] Mariotti A. (2015). The effects of chronic stress on health: new insights into the molecular mechanisms of brain–body communication. Future Sci. OA 1, FSO23. 10.4155/fso.15.21 PMC5137920 28031896

[B27] Maturana H. R. Varela F. J. (1991). Autopoiesis and Cognition: The Realization of the Living, Vol. 42. New York, NY: Springer Science and Business Media.

[B28] McEwen B. S. (2016). In pursuit of resilience: stress, epigenetics, and brain plasticity. Ann. N. Y. Acad. Sci. 1373, 56–64. 10.1111/nyas.13020 26919273

[B29] McEwen B. S. (2017). Neurobiological and systemic effects of chronic stress. Chronic Stress 1, 2470547017692328. 10.1177/2470547017692328 28856337 PMC5573220

[B30] Mousikou M. Kyriakou A. Skordis N. (2023). Stress and growth in children and adolescents. Horm. Res. Paediatr. 96, 25–33. 10.1159/000521074 34814153

[B31] Ponzi D. Muehlenbein M. P. Geary D. C. Flinn M. V. (2016). Cortisol, salivary alpha-amylase and children's perceptions of their social networks. Soc. Neurosci. 11, 164–174. 10.1080/17470919.2015.1045988 25919481

[B32] Robles T. F. (2021). Annual research review: social relationships and the immune system during development. J. Child Psychol. Psychiatry 62, 539–559. 10.1111/jcpp.13350 33164229

[B33] Sapolsky R. M. (2004). Why *Zebras* don't *Get Ulcers*: The *Acclaimed Guide* to *Stress, Stress*-related *Diseases, and Coping*. New York, NY: Holt paperbacks.

[B34] Smith K. E. Pollak S. D. (2021). Social relationships and children's perceptions of adversity. Child Dev. Perspect. 15, 228–234. 10.1111/cdep.12427 35873906 PMC9291150

[B35] Svenaeus F. (2022). Health and illness as enacted phenomena. Topoi 41, 373–382. 10.1007/s11245-021-09747-0 34024965 PMC8131875

[B36] Trevarthen C. Delafield-Butt J. Dunlop A. W. (Eds.). (2018). The Child's Curriculum: Working with the Natural Values of Young Children. Oxford: Oxford University Press. 10.1093/oso/9780198747109.001.0001

[B37] Trianes M. V. Mena M. J. B. Fernández-Baena F. J. Escobar M. Maldonado E. F. (2011). IECI. Inventario de *Estrés Cotidiano Infantil*. Madrid: TEA Ediciones.

[B38] Valsiner J. (2017). A Guided Science: History of Psychology in the Mirror of its Making. London: Routledge. 10.4324/9781315083544

[B39] Varela F. (1996). Neurophenomenology: a methodological remedy for the hard problem. J. Conscious. Stud. 3, 330–349.

[B40] Varela F. J. Shear J. (1999). First-person methodologies: what, why, how. J. Conscious. Stud. 6, 1–14.

[B41] Varela F. J. Thompson E. Rosch E. (1991). The Embodied Mind. Cognitive Science and Human Experience. Cambridge, MA: MIT press.

[B42] Wehbe A. T. Costa T. E. Abbas S. A. Costa J. E. Costa G. E. Wehbe T. W.. (2022). The effects of the COVID-19 confinement on screen time, headaches, stress and sleep disorders among adolescents: a cross sectional study. Chronic Stress 6, 24705470221099836. 10.1177/24705470221099836 35574178 PMC9096190

